# On the importance of root traits in seedlings of tropical tree species

**DOI:** 10.1111/nph.16370

**Published:** 2020-01-31

**Authors:** Coline C. F. Boonman, Frank van Langevelde, Imma Oliveras, Jeremy Couédon, Natascha Luijken, David Martini, Elmar M. Veenendaal

**Affiliations:** ^1^ Environmental Science Radboud University 6525 AJ Nijmegen the Netherlands; ^2^ Plant Ecology and Nature Conservation Group Wageningen University 6700 AA Wageningen the Netherlands; ^3^ Wildife Ecology and Conservation Group Wageningen University 6700 AA Wageningen the Netherlands; ^4^ School of Life Sciences Westville Campus University of KwaZulu‐Natal Durban 4000 South Africa; ^5^ Change Institute School of Geography and the Environment University of Oxford Oxford OX1 3QY UK; ^6^ Department of Biogeochemical Integration Max Planck Institute for Biogeochemistry Hans‐Knöll‐Straße 10 Jena 07745 Germany

**Keywords:** biomass allocation, rooting depth, root morphology, specific root length, tropical forest, savanna, vertical root distribution

## Abstract

- Plant biomass allocation may be optimized to acquire and conserve resources. How trade‐offs in the allocation of tropical tree seedlings depend on different stressors remains poorly understood. Here we test whether above‐ and below‐ground traits of tropical tree seedlings could explain observed occurrence along gradients of resources (light, water) and defoliation (fire, herbivory).
- We grew 24 tree species occurring in five African vegetation types, varying from dry savanna to moist forest, in a glasshouse for 6 months, and measured traits associated with biomass allocation.
- Classification based on above‐ground traits resulted in clusters representing savanna and forest species, with low and high shoot investment, respectively. Classification based on root traits resulted in four clusters representing dry savanna, humid savanna, dry forest and moist forest, characterized by a deep mean rooting depth, root starch investment, high specific root length in deeper soil layers, and high specific root length in the top soil layer, respectively.
- In conclusion, tree seedlings in this study show root trait syndromes, which vary along gradients of resources and defoliation: seedlings from dry areas invest in deep roots, seedlings from shaded environments optimize shoot investment, and seedlings experiencing frequent defoliation store resources in the roots.

## Introduction

Plant species' performance is greatly dependent on resource management – the balance between acquisition and conservation. The functional equilibrium hypothesis (Brouwer, 1963) states that the optimization of resource acquisition is achieved by allocating biomass to the plant organ that takes up the most limiting resource. For example, to overcome light limitation, the allocation of biomass to stem is increased to promote growth towards direct sunlight (e.g. Poorter *et al.*, 2012). Likewise, the allocation of biomass to leaves is increased to optimize light capture in shaded environments (Freschet *et al.*, 2015). However, in tropical forests the direct allocation to above‐ground plant organs that acquire limiting above‐ground resources is predicted to reduce biomass allocation towards below‐ground plant organs. However, in this environment, roots must still provide sufficient water and nutrients to support above‐ground structures, requiring high fine‐root length at depths with high supply of resources (e.g. Ansley *et al.*, 1991; Mou *et al.*, 1997; Comas *et al.*, 2002; Hoffmann *et al.*, 2004; Roumet *et al.*, 2006; Wang *et al.*, 2006). Such high‐density fine‐root structures are thus a necessary adaptation for rapid nutrient acquisition to support leaf production, transpiration and maintenance in wet tropical regions where nutrient recycling is high (e.g. Vitousek & Sanford, 1986; Coomes & Grubb, 2000; Markesteijn & Poorter, 2009). However, extensive fine‐root structures are only feasible when a stable sufficient moisture regime exists (Sainju & Good, 1993; Tyree *et al.*, 1998; Laio* et al.*, 2006). In drier areas, where water is the limiting resource as evaporative demand exceeds the amount of precipitation, plants require increased below‐ground biomass allocation to form long roots that reach deeper available soil water in order to satisfy water demand (Canadell *et al.*, 1996; Nicotra *et al.*, 2002; Schenk & Jackson, 2002; Laio *et al.*, 2006; Markesteijn & Poorter, 2009; Poorter *et al.*, 2012). This investment should come at the cost of shoot biomass.

Plants in tropical systems may encounter not only resource limitation (i.e. water and light), but also defoliation by herbivores or fire. Defoliation frequency and intensity differ along a gradient of vegetation types, mainly peaking in humid savannas at intermediate levels of rainfall (Hempson *et al.*, 2015). To enable regrowth after defoliation, plants may adjust their biomass allocation, investing more in root systems (Tomlinson *et al.*, 2012). Here, resource conservation plays a major role, especially when trees need to survive under relatively dry conditions in combination with herbivory or fire. The global trend of increased below‐ground biomass allocation with decreasing precipitation (Nicotra *et al.*, 2002; Poorter *et al.*, 2012) is not always found in plants occurring in humid savannas. Here, the vegetation deals with regular dry periods and is simultaneously frequently disturbed by herbivory or fire. These conditions may require investment in root carbon storage for fast regrowth rather than investment in rapid root development to deeper layers as in drier savannas (Tomlinson *et al.*, 2012; Issifu *et al.*, 2019). Thus, while the functional equilibrium hypothesis predicts biomass allocation in the face of limiting resources alone, it may not correctly predict biomass allocation when other stressors are at play. How the biomass allocation trade‐offs depend on both abiotic and biotic stressors in tropical tree seedlings remains poorly understood.

**Figure 1:**
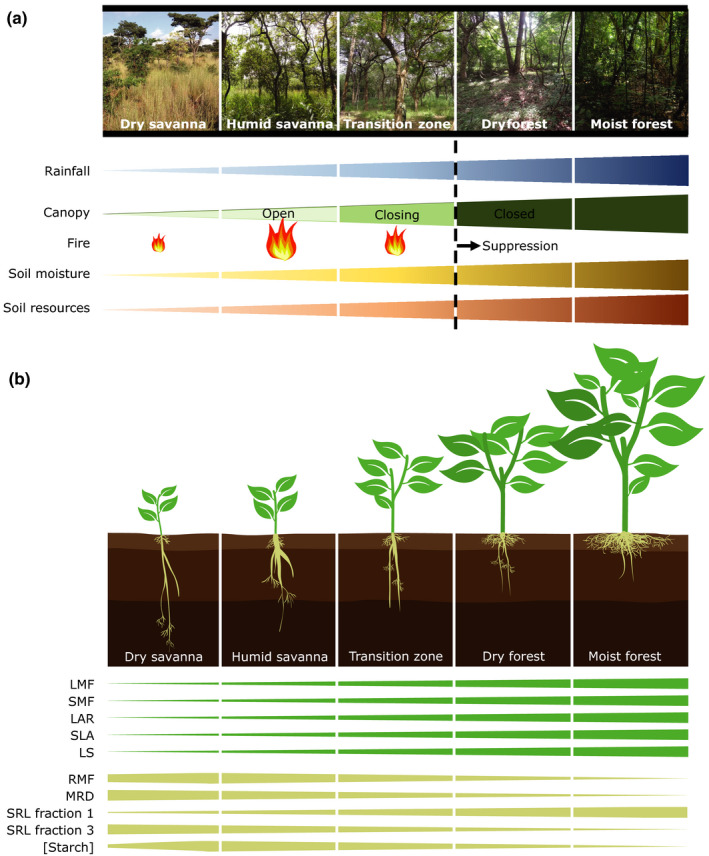
Overview of the vegetation types that are represented in this study with various environmental gradients (a), and the hypotheses for above‐ground and below‐ground traits (b). Abbreviations and descriptions of traits can be found in Table 1.

Here, we quantify morphological traits important for resource acquisition and conservation of tropical tree seedlings that cover a range of environmental gradients (from dry open vegetation to wet closed vegetation). We test for trait differences between species clusters and discuss possible trait adaptations in the context of environmental differences among vegetation types. Finally, we consider the prospect of a root economics spectrum. For this, we selected 24 tree species, which are all currently found in Africa, that are representative of a range of different vegetation types (dry savanna, humid savanna, transition zone, dry forest and moist forest) covering a gradient of different stressors (Fig. 1a; White, 1983). The three main pressures are: water limitation, supposedly the main stressor in semi‐arid tropical systems, especially in savannas (e.g. Sankaran *et al.*, 2005); light limitation, greatest in closed canopy systems such as dry and moist forest (e.g. Veenendaal *et al.*, 1996a; Markesteijn & Poorter, 2009); and defoliation frequency and intensity, peaking in humid savannas (e.g. Menaut *et al.*, 1990; Van Langevelde *et al.*, 2003).

We performed a common garden glasshouse experiment expecting the selected tree species to show intrinsic trait differences, originating from long‐term environmental filtering and/or evolutionary processes, which allow species to persist that environment. In accordance with the functional equilibrium hypothesis, we hypothesized that tree seedlings grown under resource limitation allocate biomass to where the limiting resource is located in order to maintain or increase plant growth. Furthermore, we hypothesized that defoliation stress is superior to resource limitation, and thus that seedlings from vegetation types with high levels of defoliation have traits that prioritize resource conservation to enable regrowth. Accordingly, we expected that:

1. Plants growing in drier areas have increased biomass allocation to roots, enabling a deeper mean rooting depth used to reach water from deeper soil layers as the soil profile may dry up intermittently. To enable water transport from deeper soils, a root system with long taproots is needed and thus expected.
2. Plants growing in light‐limited areas have increased biomass allocation to shoot and leaves in order to capture available light while reducing maintenance.
3. To afford these above‐ground structures in light‐limited areas, a large fine‐root length is expected in the top soil layer, where in tropical forest nutrients are rapidly recycled. Such root structures can support sufficient water and nutrient uptake with minimal energy investment and a reduced rooting depth.
4. Plants growing in more frequently defoliated systems (e.g. fire‐prone humid savannas) must conserve resources and thus the roots must exhibit storage functions. This is hypothesized to result in a small mean rooting depth and a high root mass fraction. Figure 1(b) depicts these hypotheses for each considered vegetation type and for all traits that were measured in this study with parameters defined in Table 1.

**Table 1:** Trait overview with abbreviations, units, equations, and specifications of the measurements or equations.

Trait	Abbreviation	Unit	Equation	Specifications
Leaf mass fraction	LMF	–	$\frac{\text{leaf}\;\text{biomass}}{\text{total}\;\text{biomass}}$	Dry weight of all leaves (g) and the entire plant (g)
Stem mass fraction	SMF	–	$\frac{\text{stem}\;\text{biomass}}{\text{total}\;\text{biomass}}$	Dry weight of stem (g) and the entire plant (g)
Leaf size	LS	cm^2^	–	Single leaf including petiole
Specific leaf area	SLA	cm^2^ g^−1^	$\frac{\text{LS}}{\text{leaf}\;\text{weight}}$	Dry weight of leaf used for LS
Leaf area ratio	LAR	cm^2^ g^−1^	$\frac{\text{SLA}\times \text{leaf}\;\text{biomass}}{\text{total}\;\text{biomass}}$	Dry weight of all leaves and entire plant
Root mass fraction	RMF	g g^−1^	$\frac{\text{root}\;\text{biomass}}{\text{total}\;\text{biomass}}$	Dry weight of roots (g) and the entire plant (g)
Mean rooting depth	MRD	cm	$\frac{\sum_{i=1}^{4}{\text{biomass}}_{i}\times d_{i}}{\sum_{i=1}^{4}{\text{biomass}}_{i}}$	*i* is the section number *d* is mean depth per *i* (cm)
Specific root length fraction	SRL fraction	–	$\frac{{\text{SRL}}_{i}}{\sum_{i=1}^{4}{\text{SRL}}_{i}}$	*i* is the section number $\text{SRL}=\frac{\text{root}\;\text{length}}{\text{root}\;\text{weight}}\left(\frac{\text{mm}}{g}\right)$
Starch concentration	[Starch]	%	$\frac{\text{Starch}\;\text{mass}}{\text{root}\;\text{mass}}\times 100$	% by weight

## Materials and Methods

### Species selection

A total of 24 tropical tree species found in different ecosystems on the African continent (most indigenous, some introduced) were selected to use in this glasshouse common garden experiment (Table 2). Species were a priori grouped into different vegetation types based on the occurrence of natural species (Table 2). The selected species represent a gradient from dry savanna to moist forest vegetation, and include the three taxa, Meliaceae, Leguminosae, and Combretaceae, with representatives balanced across the gradient. Seeds of all selected species were collected in different vegetation types across the gradient in West Africa or, as our access to the dry savanna in the Sahelian zone was restricted, from southern Africa.

**Table 2:** Ecological information of the species used in this study (country of collection: BO, Botswana; GH, Ghana; SA, South Africa).

Species	Taxonomic guild	Main vegetation type^a^	Leaf habit of mature trees^a^	Collection location – Original distribution	Maximum height (m)^a^	Potential rainfall range (mm)^b^	Mean annual rainfall collection site	Ecological Rose and guild^b^
*Afzelia africana *Pers.	Leguminosae – Caesalpinioideae	Transition	Deciduous	Kogyae SNR GH. – West Africa	35	1200–1800	1350	Forest/Transition nonpioneer light‐demander
*Albizia lebbeck *(L.) Benth.	Leguminosae – Mimosoideae	Dry forest	Semi‐evergreen	Tamale GH. – South Asia	20	500–2500	1090	Dry forest, invasive in savanna
*Cedrela odorata *(L.)	Meliaceae	Moist forest	Deciduous	Pra Annum F.R. GH. – South America	60	1000–3700	1680	Forest pioneer
*Ceiba pentandra *(L.) Gaertn	Bombaceae	Dry forest	Deciduous	Tamale GH. – Pan‐Tropical	60	750–3000	1090	Forest pioneer
*Colophospermum mopane *(Benth.) Leonard	Leguminosae – Caesalpinioideae	Dry savanna	Deciduous	Maun BO. – Southern Africa	25	200–800	450	Savanna
*Combretum hereroense *Schinz.	Combretaceae	Dry savanna	Deciduous	Limpopo Prov. SA. – Southern Africa	23	250–750	570	Savanna
*Detarium microcarpum *Guill. & Perr.	Leguminosae – Caesalpinioideae	Humid savanna	Deciduous	Kogyae SNR GH. – West Africa	10	600–1350	1350	Savanna
*Khaya anthotheca *(Welw.) C.DC.	Meliaceae	Dry forest	Evergreen	Mpraeso GH – West Africa	65	1200–1800	1680	Forest, nonpioneer light‐demander
*Khaya grandifoliola *C.DC.	Meliaceae	Dry forest	Deciduous	Aboma GH – West Africa	40	1200–1800	1600	Forest, nonpioneer light‐demander
*Khaya senegalensis *(Desv. A. Juss.)	Meliaceae	Transition	Semideciduous	Kogyae SNR GH. – West Africa	30	400–1750	1350	Savanna
*Millettia thonningii *(Schum. & Thonn.) Baker	Leguminosae – Papilionoideae	Transition	Deciduous	Kumasi GH. – West Africa	20	600–1200	1680	Dry forest, savanna
*Nauclea diderrichii *(De Wild.) Merr.	Rubiacaeae	Moist forest	Evergreen	Kumasi GH – West Africa	40	1600–3000	2300	Forest pioneer
*Peltophorum africanum *Sond.	Leguminosae – Caesalpinioideae	Dry savanna	Deciduous	Limpopo Prov. SA. – Southern Africa	10	300–900	570	Savanna
*Pithecellobium dulce *(Roxb.) Benth.	Fabaceae – Mimosoideae	Dry forest	Evergreen	Kumasi GH. – Central America	15	250–1775	1680	Dry forest, invasive
*Prosopis africana *(Guill. & Perr.) Taub.	Fabaceae – Mimosoideae	Humid savanna	Deciduous	Kogyae SNR GH. – West Africa	20	600–1500	1350	Savanna
*Pterocarpus erinaceus *Poir.	Leguminosae – Caesalpinioideae	Humid savanna	Deciduous	Mole N.P. – West Africa	15	600–1200	1100	Savanna
*Senna siamea *(Lam.) H.S.Irwin & Barneby	Leguminosae – Caesalpinioideae	Transition	Evergreen	Kumasi GH – Asia	18	400–2800	1680	Dry forest and transition, invasive
*Terminalia glaucescens *Planch. Ex Benth	Combretaceae	Humid savanna	Deciduous	Kogyae GH. – West Africa	20	600–1400	1350	Savanna
*Terminalia ivorensis *A.Chev.	Combretaceae	Moist forest	Deciduous	Bobiri F.r. – West Africa	46	1250–3000	2125	Forest pioneer
*Terminalia schimperi *(Hochst. Ex Hutch. & Dalziel)	Combretaceae	Transition	Evergreen	Kogyae GH. – West Africa	30	200–1400	1350	Savanna and transition
*Terminalia superba *Engl. & Diels	Combretaceae	Moist forest	Deciduous	Kumasi GH – West Africa	50	1000–3000	1680	Forest pioneer
*Tetrapleura tetraptera* (Schum. & Thonn.) Taub.	Leguminosae – Mimosoideae	Moist forest	Deciduous	Kumasi GH – West Africa	25	1000–2500	1680	Forest pioneer
*Vachellia erioloba *(E.Mey.) P.J.H.Hurter	Leguminosae – Mimosoideae	Dry savanna	Deciduous	Limpopo Prov. SA. – Southern Africa	18	250–1000	570	Savanna
*Vachellia tortilis *(Forssk.) Galasso & Banfi	Leguminosae – Mimosoideae	Dry savanna	Deciduous	Limpopo Prov. SA. – Pan African Dist.	21	100–1000	570	Savanna

a *Source:* Keay (1989), Hawthorne (1995), Hawthorne & Jonkind (2006).

b *Source:* Orwa *et al.* (2009). Coordinates (longitude, latitude): Kogyae −1.16, 7.32; Tamale −0.85, 9.44; Pra annum −1.18, 6.22; Maun 23.40, −19.99; Limpopo 29.50, −23.41; Mpraeso −0.69, 6.65; Aboma −1.45, 7.18; Kumasi −1.52, 6.71; Mole −1.86, 9.25; Bobiri −1.35, 6.70.

### Experimental design

The experiment started in June 2015 and was conducted in a glasshouse of Unifarm, Wageningen University, the Netherlands. A fully randomized block design was set up with 10 blocks and a minimum of four individuals per species per block. All seedlings were raised from seeds, 6 wk before the start of the experiment. At the start of the experiment, seedlings were planted in plastic tubes 10 in diameter and 100 cm in length, filled with sand and 5 mg cm^−3^ slow‐release fertilizer (Osmocote 18 : 6 : 12 (N : P : K) fertilizer (8–9 month mixture)) removing nutrient limitation (Tomlinson *et al.*, 2012, 2013). All plants were harvested 6 months after the start of the experiment, which is about the length of a growing season in the moist savannas of West Africa and the dry savannas of southern Africa. Our analysis specifically focused on tree seedlings because this is a vulnerable life‐stage where traits determine survival in relation to stress (Stohlgren *et al.*, 1998).

In the glasshouse, relative humidity was kept at 80%, while temperature changed every 12 h from 28 to 23°C during the day and night, respectively. Natural daylight (60–70% of outside light) was allowed into the glasshouse for 12 h daily with a minimum photon flux density of 200 μmol s^−1^ m^−2^ during dark daytime conditions ensured by extra lightning. Half of the seedlings received 45 ml and the other half 90 ml of water per day, aimed to be representative of a humid savanna and semideciduous forest rainy season precipitation, respectively (Veenendaal *et al.*, 1996b). However, this treatment level was not severe enough to cause differences between plants and has therefore been omitted from further analyses (results not shown). Although some species in this experiment originated from drier conditions, we considered the prevention of water stress in water‐demanding species as being more important.

### Harvest and processing of plant material

Individuals were harvested and separated into roots, stem and leaves. Stem length was measured, and total and single leaf area were determined by scanning a representative subsample with an LI‐3100 leaf area meter (Li‐Cor, Lincoln, NB, USA). The roots were carefully rinsed with tap water, and divided with depth in up to four 25 cm sections to determine vertical root biomass distribution. A subsample, the middle 5 cm of each section, was used for a root scan to describe vertical fine‐root length distribution. This subsample was scanned at 400 dpi with an Epson Perfection V800 and analyzed with winrhizo software (Regent Instruments, Québec, QC, Canada; Arsenault *et al.*, 1995). The other 20 cm of root material per section was microwaved at 800 W for 40–120 s in order to kill cells to avoid further starch consumption.

After processing, all leaf, stem and root material of each plant was separately dried for 72 h at 65°C and weighed to determine biomass allocation to the respective parts and sections. Root starch content was extracted and measured for each individual after fine grinding the dried root material from the section closest to the surface (0–25 cm depth; Duranceau *et al.*, 1999; Cardoso *et al.*, 2016).

### Traits

We considered five above‐ground and five below‐ground traits that describe biomass allocation and are important for resource acquisition or resource conservation: leaf mass fraction, stem mass fraction, leaf size, specific leaf area, leaf area ratio, root mass fraction, mean rooting depth, specific root length fraction for the top section (0–25 cm depth) and for the third section (50–75 cm depth), and starch content (Table 1). The selected above‐ground traits were chosen to represent biomass distribution to the above‐ground plant organs. Leaf mass fraction (LMF) and stem mass fraction (SMF) show the broad biomass partitioning, while leaf size (LS, cm^2^), specific leaf area (SLA, cm^2^ g^−1^) and leaf area ratio (LAR, cm^2^ g^−1^) indicate the partitioning to leaf functioning. The selected below‐ground traits were chosen to gain an understanding of the root architecture. Because plants were grown in pipes, we focused on the vertical below‐ground biomass distribution. Root mass fraction (RMF) shows the broad biomass partitioning to below‐ground organs, while mean rooting depth (MRD, cm) calculates the depth of mean biomass investment (Mommer *et al.*, 2010). The specific root length (SRL) fractions for sections 1 (0–25 cm) and 3 (50–75 cm) specify root investment at different depths. We used fractional data, as we expected the general root investment to differ per species. To gain an understanding of the distribution of root investment over the whole root system, we compared SRL fractions of each section with an SRL value of 0.25, which would occur if there is equal investment in root structure over the full length of the root. When the SRL fraction is lower than 0.25, there is more biomass investment in that specific root section. This means that there are thicker roots in that section compared to the other sections. Starch content was measured to indicate a level of resource conservation.

### Statistical analysis

To determine whether above‐ or below‐ground traits can explain the observed occurrences of the selected tree species over the vegetation types, we used a hierarchical clustering technique with Euclidean distances and complete linkage (*hclust* function in ‘stats’ package; R Core Team, 2016). Thus, the resulting clusters are based on species‐level trait differences, and differences between clusters are indicated by branch length, where longer branches indicate larger trait differences between species. This clustering technique assumes no correlations among the different input variables. Because the selected plant traits were correlated (Supporting Information Table S1), we ran two principal components analyses (PCAs) and used the PCA scores as input for the clustering instead of the original trait values.

On the basis of the identified clusters, we ran a linear mixed model for each trait to determine trait differences among clusters (*lmer* function in ‘lme4’ package; Bates *et al.*, 2015). Per model, one trait was selected as the response variable, ‘cluster’ was the fixed effect, and species and blocks from the experimental design were random effects. When ‘cluster’ appeared to be significant in the model, we performed a Tukey post‐hoc test to identify the differences between clusters. Differences in vertical root biomass distribution (SRL fractions 1 and 3) from the equal distribution null hypothesis (SRL fraction = 0.25) were determined by performing one‐sample *t*‐tests.

To check if species clusters represent differences in environmental variables, we ran a linear model with ‘cluster’ as a fixed effect. The chosen response variables were average annual rainfall (mm) as a proxy for water limitation, normalized difference vegetation index (NDVI images of MODIS (1 month‐Terra) from the NEO Nasa Earth Observations) as a proxy for vegetation cover and light limitation, fire frequency as a proxy for defoliation pressure (number of fires per 1000 km^2^ yr^−1^), and soil cation exchange capacity averaged over the top 30 cm (CEC; cmol^+^ kg^−1^) as a proxy for soil resources (Veenendaal *et al.*, 2015). These data were obtained from global maps (Hengl *et al.*, 2014; Karger *et al.*, 2017; NASA Earth Observations, 2017) using the location of the seed collection per species (Table 2).

For all analyses, data were transformed when necessary to meet the assumptions of the statistical tests, this being indicated with each result. All analyses were conducted using r v.3.4.1 (R Core Team, 2016).

## Results

**Figure 2:**
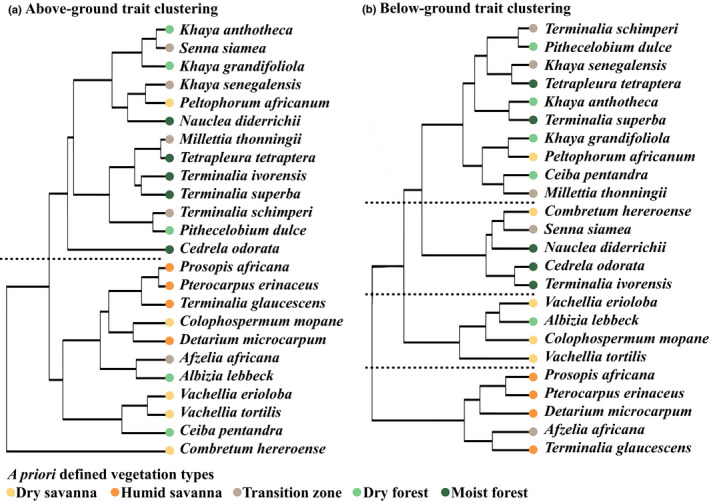
Clustering of species by (a) above‐ground traits and (b) below‐ground traits.

**Figure 3:**
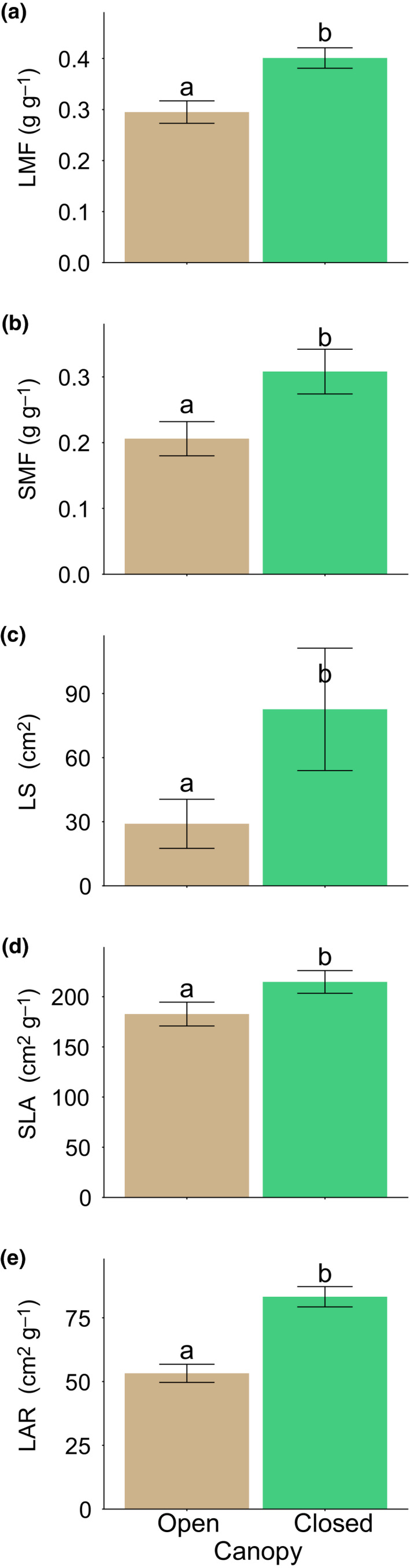
Above‐ground trait averages of the species clusters as identified in Fig. 2(a). Bars represent marginal means and error bars show ± SE for (a) leaf mass fraction (LMF), (b) stem mass fraction (SMF), (c) leaf size (LS), (d) specific leaf area (SLA) and (e) leaf area ratio (LAR). Lower‐case letters indicate significant differences (*P* < 0.05). *Combretum hereroense* was excluded from this analysis, as it did not fit either of the two species clusters.

Clustering species based on above‐ground traits resulted in two main clusters (Fig. 2a), with possible alternatives considered (Notes S1; Figs S1, S2): one with mainly dry and moist savanna species representing an open canopy vegetation, and one with transition and forest species representing a closing and closed canopy vegetation. The clusters showed significant differences in all traits measured (Fig. 3; Tables S3, S4). The open canopy vegetation cluster had significantly lower values in all above‐ground traits compared to the closed or closing canopy vegetation cluster: LMF 0.30 ± 0.2 vs 0.40 ± 0.02 (marginal mean ± SE), SMF 0.21 ± 0.03 vs 0.31 ± 0.03, LS 29 ± 11.5 vs 82.6 ± 28.6 cm^2^, SLA 183 ± 11.8 vs 215 ± 11.3 cm^2^ g^−1^ and LAR 53.2 ± 3.6 vs 83.2 ± 4.0 cm^2^ g^−1^. Trait values of individual species and model results are given in Tables S2 and S3, respectively.

**Figure 4:**
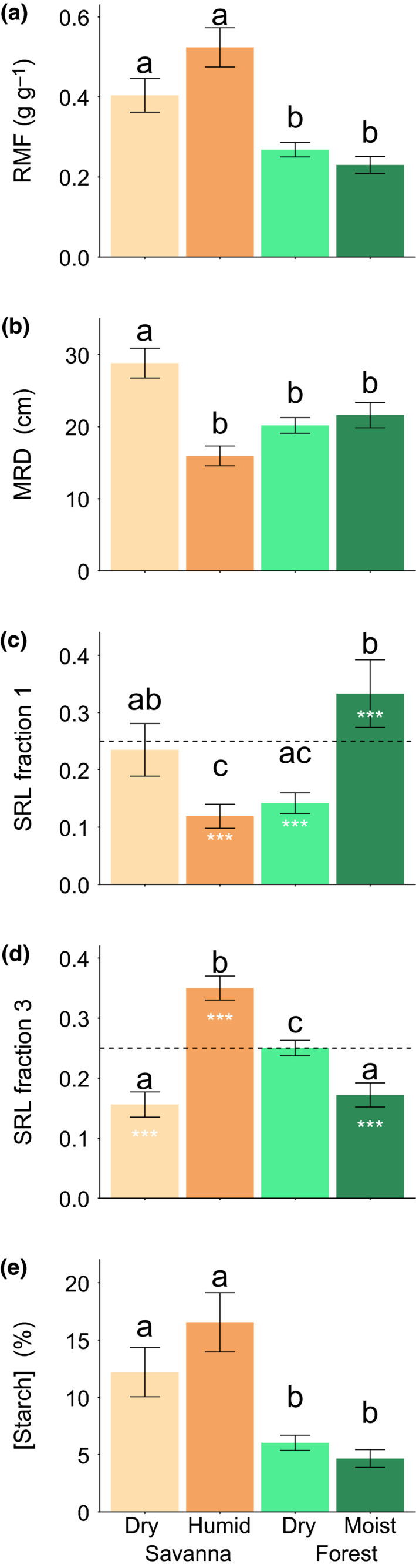
Below‐ground trait averages of species clusters as identified in Fig. 2(b). Bars represent marginal means and error bars show ± SE for (a) root mass fraction (RMF), (b) mean rooting depth (MRD), (c) specific root length fraction for the uppermost section of the root (SRL fraction 1), (d) specific root length fraction for a deeper section of the root (SRL fraction 3) and (e) starch concentration, ([Starch]). Lower‐case letters indicate significant differences between species clusters (*P* < 0.05). The dashed line in (c) and (d) represents the null hypothesis of equal distribution of fine roots over the whole length of the root, where asterisks indicate significant differences with this null hypothesis (*P* < 0.05).

Clustering species based on below‐ground traits resulted in four clusters (Fig. 2b), with possible alternatives considered (Notes S1; Figs S1, S2). One was dominated by dry savanna species, one by humid savanna species, one by species usually occurring in dry forest and one by mostly species from moist forests. Species *a priori* defined to originate in transition zones were mostly associated with the cluster with dry forest species and the cluster with humid savanna species. The dry savanna cluster was characterized by a greater MRD (28.8 ± 2.06 cm), a large RMF (0.40 ± 0.04), an intermediate specific root length at shallow depth (SRL fraction 1 = 0.24 ± 0.05), a significantly lower specific root length at deeper depth than the equal distribution null hypothesis of 0.25 (SRL fraction 3 = 0.16 ± 0.02) and a high starch content (12.2 ± 2.1%; Fig. 4; Tables S3, S4). The humid savanna cluster was characterized by a smaller MRD (15.9 ± 1.37 cm), a large RMF (0.52 ± 0.05), a lower SRL fraction 1 than the equal distribution null hypothesis (0.12 ± 0.02), a higher SRL fraction 3 than the equal distribution null hypothesis (0.35 ± 0.02) and a high starch content (16.6 ± 2.6%) (Fig. 4; Tables S3, S4). The dry forest cluster was characterized by an intermediate MRD (20.2 ± 1.09 cm), a smaller RMF (0.27 ± 0.02), a lower SRL fraction 1 than the equal distribution null hypothesis (0.14 ± 0.02), an intermediate SRL fraction 3 (0.25 ± 0.01) and a lower starch content (6.0 ± 0.7%) (Fig. 4; Tables S3, S4). The moist forest cluster was characterized by an intermediate MRD (20.6 ± 1.56 cm), a smaller RMF (0.23 ± 0.02), a higher SRL fraction 1 than the equal distribution null hypothesis (0.33 ± 0.06), a lower SRL fraction 3 than the equal distribution null hypothesis (0.17 ± 0.02) and a lower starch content (4.7 ± 0.8%) (Fig. 4; Tables S3, S4).

Clusters obtained from above‐ and below‐ground traits reflected differences in fire frequency, and water, light and nutrient availability (Fig. S3). As hypothesized, fire frequency and light availability were higher while water availability was lower for the savanna clusters than for the forest clusters (Fig. S3). However, contrasting with our hypothesis, soil CEC values were higher for the savanna clusters than for the forest clusters (Fig. S3).

## Discussion

Our common garden experiment describes above‐ and below‐ground trait variation of tropical tree seedlings representative of a range of different vegetation types with gradients of resource limitation and defoliation. We aimed in particular at describing intrinsic differences in traits that were chosen to assess trade‐offs in response to combinations of abiotic and biotic stressors encountered in the field. We acknowledge the limitations of a glasshouse experiment in which, for instance, spatially restricted potting pipes influence rooting patterns and thus trait values. Our trait values are therefore likely to differ from those found in unrestricted soil columns. In addition, we did not test the plasticity of traits, which is commonly observed both in the field and in glasshouses when light or soil conditions vary (e.g. Veenendaal *et al.*, 1996a,c; Schenk & Jackson, 2002; Markesteijn & Poorter, 2009; Tomlinson *et al.*, 2012, 2013; Wigley *et al.*, 2019). Nevertheless, as our experiment was conducted under the same controlled environmental conditions for all species, any trait differences observed should strongly suggest significant, evolutionarily driven intrinsic trait differences.

### Species clustering

We found that leaf traits could only distinguish between two broad vegetation types, namely open canopy savanna vegetation and closed or closing canopy forest vegetation, while root traits could differentiate between four vegetation types, namely the drier and the more humid type of savanna and forest. The clusters largely reflected the vegetation types as defined for each species. However, species from the same origin did not always end up in the same cluster (Fig. 2). An example is the dry savanna species *Combretum hereoense*, which was dissimilar to the two above‐ground trait‐based clusters and was placed in the cluster with moist forest species in the below‐ground trait‐based clustering (Fig. 2). *C. hereoense* originates from drier areas, but is associated with cool rocky hill sides, riverine woodland and regularly flooded areas (Coates‐Palgrave, 1984; Siebert *et al.*, 2002). This illustrates that even within a vegetation type, a great diversity of environmental conditions and thus trait variation can still exist. Another example is the dry forest species *Albizia lebbeck*. It was clustered with savanna species, which corresponds with the site where our provenance was collected (Table 2). This indicates that genotype variation within species may affect results (e.g. Vandenbelt, 1991) – especially for species with a wide ecological amplitude such as *Albizia lebbeck* (Table 2) – and reflects that our provenance represents a drier genotype of this genetically variable species (Toky, 2000). Regardless of such causes of trait variation, our clustering results are remarkably robust. The clusters reflect differences in rainfall, fire frequency and vegetation cover (i.e. NDVI) (Fig. S3). Relationships with soil fertility of the substrate (with soil CEC as a proxy) are more complex. Soil CEC did not increase with increasing vegetation cover as hypothesized (Fig. 1). In West Africa, more fertile soils are often found in drier forest and savanna transitions, and poorer, more leached soils with lower CEC in forests that experience a wetter climate (e.g. Swaine, 1996), illustrating the interaction between climate, soils and vegetation over longer time scales. Relationships between natural vegetation and soil resources must also be interpreted cautiously due to the low fidelity of global soil data presently available (Moulatlet *et al.*, 2017). In tropical forests, nutrient fluxes will mostly take place at the soil surface with increasing tree cover through recycling of litter, irrespective of the nutrient status of the substrate, causing increased fluxes and availability of nutrients as implied in Fig. 1 (e.g. Vitousek & Sanford, 1986). Increased vegetation cover is also directly linked to increased fine root production and, by implication, competitive pressure for available nutrients (Chen *et al.*, 2019).

### Traits per cluster

From the clusters obtained by above‐ground trait clustering, the closed or closing canopy cluster showed an increased biomass investment in leaf (LMF) and stem (SMF) compared to the open canopy cluster, presumably to optimize photosynthetic ability under light‐limited conditions (Figs 3, S3). These patterns are in line with the functional equilibrium hypothesis and confirm previous studies on above‐ground trait differences between savanna and forest species (e.g. Veenendaal *et al.*, 1996b; Hoffmann & Franco, 2003; Poorter *et al.*, 2012; Ratnam *et al.*, 2019). These patterns also translate to root biomass investment (RMF), where species in the closed or closing canopy forest clusters allocate less biomass to roots than the species in the open canopy savanna clusters (Fig. 4a). The higher root investment found in the savanna clusters could be explained by drought and defoliation being the main stressor (Fig. S3).

We also observed other meaningful below‐ground trait differentiation between the four clusters obtained by below‐ground trait clustering (Fig. 4). The dry savanna cluster was distinct with, for example, a larger amount of biomass in deeper soil layers (MRD and SRL fraction 3; Fig. 4b,d), indicative of a more persistent, robust taproot system needed for exploration of soil water under conditions of irregular rainfall or a prolonged dry season (e.g. Aref, 1996; Wilson & Witkowski, 1998; Padilla & Pugnaire, 2007). Additionally, species in this cluster had increased root starch levels (Fig. 4e), which is hypothesized to be needed to compensate for sprout loss through drought, fire or herbivory (e.g. Schutz *et al.*, 2009). The humid savanna cluster had thick roots near the soil surface (MRD, SRL fraction 1) with a storage function reflected by the high starch content (Fig. 4b,c,e; Cardoso *et al.*, 2016; Gignoux *et al.*, 2016; Issifu *et al.*, 2019). Species from this cluster also rooted less deeply (Fig. 4b), which confirms our hypothesis of the superior effect to overcome defoliation over predictions based on the functional equilibrium hypothesis to acquire resources. The dry forest cluster had thick roots near the soil surface (SRL fraction 1), but without the starch storage function of the species from the humid savanna cluster (Fig. 4c,e). Species in this cluster showed larger fine‐root length at deeper depths (SRL fraction 3), probably adjusted to seasonal drier periods when roots explore moisture from deeper soils instead of close to the surface (Fig. 4d). Species from the moist forest cluster also showed an adjusted root system, with a well‐developed fine‐root system in the top soil layer (SRL fraction 1; Fig. 4c), which makes for a structure that is limited in terms of biomass while being very effective for rapid water and particularly nutrient uptake in area with increased fine root densities (Sanford, 1989; Coomes & Grubb, 2000; Prieto *et al.*, 2015). They also show an increased amount of biomass investment in deeper soil layers (SRL fraction 3; Fig. 4d), presumably to mechanically ensure stability for tall above‐ground structures.

### Tropical tree seedling root trait syndromes

Our common garden experiment confirms the hypothesis that tropical tree seedlings from different vegetation types have distinct traits, where root morphological traits are also representative of the distinction between specific sets of growth‐limiting factors or stressors – more so than above‐ground traits. Our results can be summarized in four tropical tree seedling root trait syndromes (RTSs):

1. A light‐limited RTS describes root morphological traits for tree seedlings growing in a light‐limited environment only. Seedlings have a dense fine‐root structure close to the soil surface, which enables effective water and nutrient acquisition, enabling the maintenance of sufficient leaf structures for light capture and rapid growth.
2. A water‐limited RTS describes root morphological traits for tree seedlings growing in a water‐limited environment. Seedlings have thicker, longer roots to reach moisture from deeper soil layers, while simultaneously storing starch for regrowth.
3. A defoliation RTS describes root morphological traits for tree seedlings growing in an environment where fire and/or herbivory cause frequent defoliation. Seedlings often have a storage organ in the form of a thick root where energy in the form of starch is stored, which enables regrowth after defoliation but reduces growth during early establishment.
4. A dry forest RTS describes root morphological traits for tree seedlings growing in an environment where light is limited, fires occur at a low frequency and seasonal water deficits occur. Seedlings often have roots to retain water and nutrients from deeper soil layers rather than from the top layer as in the light‐limited RTS*.*

These four RTSs for tropical tree seedlings do not cover all plant trait options (e.g. plant defence systems; Hanley *et al.*, 2007) or optimization to all combinations of abiotic and biotic stressors present. However, there are many field studies that underwrite the importance of RTSs as proposed here in areas where multiple types of stressors occur. In recent transplant studies in forest–savanna transitions the recruitment success of different tree functional types confirms an important role of our selected root traits in establishment success (Cardoso *et al.*, 2016; Issifu *et al.*, 2019).

While our RTSs are quite intuitive, and can be extracted from the literature, they have, to our knowledge, not been described in a comparative study and with this amount of detail before. We found large differences in root traits even without severe stress present in the glasshouse, which means they must have been evolutionarily evolved due to environmental filtering.

### A taste of a root economic spectrum?

One way to understand plant resource management is the fast–slow continuum: a strategy aimed at nutrient acquisition associated with fast growth and less durable structures compared to a nutrient‐conserving strategy which results in slow growth (Wright *et al.*, 2004). This fast–slow continuum has been observed for leaf and wood traits, resulting in a global leaf and wood economics spectrum, respectively (Wright *et al.*, 2004; Chave *et al.*, 2009). With the increasing recognition that roots play an important role in structuring plant communities (Comas, 2017), as also suggested by our results, the formulation of a root economics spectrum (RES) is in demand. Previous attempts failed to find a parallel with the leaf economics spectrum (LES) using fine‐root traits (Reich, 2014; Weemstra *et al.*, 2016). However, roots have more functions than just resource capture or structural support, like leaves and stem, respectively. Roots also store reserves that can be used for regrowth after defoliation (Tomlinson *et al.*, 2012). We argue that, as with the leaf and wood economic spectrum, a potential RES will be influenced by a strong filtering effect from the environment (Wiemann & Williamson, 2002; Wright *et al.*, 2004, 2017). Therefore, we suggest, when formulating an RES, including species over the full breath of environmental gradients as we did in our study. Although we only looked at morphological root traits in a relatively limited number of tree species and have not taken into account any nonwoody species, other growth forms, adult individuals or physiological root traits (e.g. Gignoux *et al.*, 2016), we suggest that an RES for tropical tree seedlings should be made on two axes. The first axis is the resource limitation axis, where water limitation stands on one end and light limitation on the other. The second axis presents the risk of defoliation. Most environments worldwide will be somewhere in the middle of the first axis, and towards zero on the second axis. In these areas, roots can generally take any form as tree seedling survival is more a matter of chance (i.e. to not be stamped, eaten by herbivores/browsers or die from disease) than it is to actually overcome any of the three growth‐limiting, and actually lethal, factors used as stressors in this study. Thus, while the LES is found globally and for any growth form, an RES for trees will probably only be visible if environmental gradients are sufficiently large. The patterns we find in our study may also apply for the sapling stage, which may experience similar stressors, such as shading, intermittent drought and seasonal defoliation. Studies by, for example, Timberlake & Calvert (1993), Gignoux *et al.* (2006) and Wigley *et al.* (2019) also suggest similarities between root architecture of seedlings and saplings, even though these studies did not quantify this in a similar manner or examined species covering the full breath of vegetation types as in our study. At the mature stage, additional root functions such as mechanical stability, extended soil volume exploration and avoidance of critical stem cavitation during prolonged droughts may be important traits leading to deviations from the discussed RES patterns (Coutts, 1983; Stone & Kalisz, 1991; Kotowska *et al.*, 2015). These later transitions are an important area for further research (see also Poorter *et al.*, 2012). In the present study, we combined differences in tree seedling biomass allocation and functional traits from a range of African tropical vegetation types to trait syndromes. In conclusion, our results demonstrate that root traits in particular differ substantially among species occurring in the different vegetation types: long deep growing roots in dry areas, root structure with many fine roots close to the soil surface in light‐limiting environments, and a storage organ when defoliation risk is high. This large variety indicates that root traits of tree seedlings are susceptible to environmental filtering, and confirms that intrinsic root traits play a large role in tree seedling survival.

## Author contributions

EMV, IO and FvL designed the study and obtained funding; EMV, IO, FvL and JC set up and ran the experiment; CCFB and EMV analyzed the data and wrote the manuscript with contributions from FvL and IO. During the experiment, CCFB, EMV, IO, FvL, NL and DM were engaged in the harvests and trait measurements. All authors were also engaged in revisions of the manuscript.

## Supporting information

Please note: Wiley Blackwell are not responsible for the content or functionality of any Supporting Information supplied by the authors. Any queries (other than missing material) should be directed to the *New Phytologist* Central Office.

**Fig. S1** Overview of different clustering options for (a) above‐ground trait clusters and (b) below‐ground trait clusters.
**Fig. S2** Trait averages of species clusters as identified in Fig. S1.
**Fig. S3** Environmental variable averages of species clusters as identified in Fig. S1.
**Notes S1** Alternative clustering options and cluster relationships to environmental variables
**Table S1** Rho values of spearman correlations between all plant traits.
**Table S2** All species trait values – mean and standard deviation.
**Table S3** General linear mixed model results for the differences between the vegetation clusters in leaf traits (savanna, forest), and root traits (dry savanna, humid savanna, dry forest, moist forest).
**Table S4** Above‐ground trait values per cluster – marginal mean and standard error; below‐ground trait values per cluster – marginal mean and standard error.
