# Supplementary material for: On the importance of root traits in seedlings of tropical tree species
Source: New Phytol. 2020 Jan 31;227(1):156–67. doi: 10.1111/nph.16370 (PMC7317509; doi:10.1111/nph.16370)
Supplement: Supplementary file 1 — Fig. S1 Overview of different clustering options for (a) above‐ground trait clusters and (b) below‐ground trait clusters. Fig. S2 Trait averages of species clusters as identified in Fig. S1. Fig. S3 Environmental variable averages of species clusters as identified in Fig. S1. Notes S1 Alternative clustering options and cluster relationships to environmental variables Table S1 Rho values of spearman correlations between all plant traits. Table S2 All species trait values – mean and standard deviation. Table S3 General linear mixed model results for the differences between the vegetation clusters in leaf traits (savanna, forest), and root traits (dry savanna, humid savanna, dry forest, moist forest). Table S4 Above‐ground trait values per cluster – marginal mean and standard error; below‐ground trait values per cluster – marginal mean and standard error. [file NPH-227-156-s001.pdf]

***New Phytologist* Supporting Information**

Article title: On the importance of root traits in seedlings of tropical tree species

Authors: Coline C.F. Boonman[1,2], Frank van Langevelde[3,4], Imma Oliveras[5], Jeremy Couédon[2], Natascha Luijken[2], David Martini[2,6], Elmar M. Veenendaal[2]

Article acceptance date: 01 December 2019

The following Supporting Information is available for this article:

Table S1 Trait correlations

Table S2 Species trait values

Table S3 Model results

Table S4 Cluster trait values

Notes S1 Alternative clustering options and cluster relationships to environmental variables

Figure S1 Overview of different clustering options

Figure S2 Trait averages of species clusters as identified in Figure S1

Figure S3 Environmental variable averages of species clusters as identified in Figure S1.

Table S1 Rho values of spearman correlations between traits. Asterisks indicate significance level of the correlation ($^{*}p<0.05$, $^{**}p<0.01$, $^{***}p<0.001$). LMF is leaf mass fraction, SMF is stem mass fraction, LS is leaf size, SLA is specific leaf area, LAR is leaf area ratio, RMF is root mass fraction, MRD is mean rooting depth, SRL fr1 is specific root length fraction of the first section of the root, SRL fr3 is specific root length fraction of the third section of the root, and [starch] is the starch concentration of the first section of the root.

	SMF	LS	SLA	LAR	RMF	MRD	SRL fr1	SRL fr3	[starch]
LMF	-0.23***	0.23***	0.07	0.72***	-0.71***	-0.1	0.19**	-0.09	-0.54***
SMF		0.15**	0.08	-0.09	-0.43***	0.27***	0.04	-0.17*	-0.19**
LS			-0.05	0.11*	-0.37***	-0.13	0.05	-0.06	-0.31***
SLA				0.71***	-0.09	0.08	-0.01	0.02	-0.2**
LAR					-0.52***	-0.01	0.14*	-0.02	-0.49***
RMF						-0.02	-0.16*	0.13	0.63***
MRD							0.13	-0.4***	-0.1
SRL fr1								-0.53***	-0.19**
SRL fr3									0.24***

Table S2 Species trait values (mean and standard deviation). Plant weight is the total biomass of the plant, LMF is leaf mass fraction, SMF is stem mass fraction, LS is leaf size, LAR is leaf area ratio, SLA is specific leaf area, RMF is root mass fraction, MRD is mean rooting depth, SRL fr1 is specific root length fraction of the first section of the root, SRL fr3 is specific root length fraction of the third section of the root, and [starch] is the starch concentration of the first section of the root. This data is also available in TRY: dataset ID 695, name Above.and.Below.ground.tree.seedling.traits.

Species	plant weight	LMF	SMF	LS	SLA	LAR	RMF	MRD	SRL fr1	SRL fr3	[starch]
Afzelia africana	32 - 13.2	0.29 - 0.11	0.24 - 0.07	144.3 - 87.6	193.4 - 46.6	54.9 - 23.6	0.47 - 0.08	19 - 1.9	0.12 - 0.05	0.4 - 0.15	12.3 - 4.3
Albizia lebbeck	92.9 - 45.2	0.27 - 0.09	0.25 - 0.07	288.2 - 136.4	222.8 - 65.8	63.5 - 30.6	0.48 - 0.13	26.4 - 2.5	0.24 - 0.11	0.14 - 0.04	13.2 - 5.7
Cedrela odorata	90.3 - 49.9	0.37 - 0.1	0.32 - 0.08	465 - 295.8	259.1 - 47.9	95.5 - 29.6	0.3 - 0.09	17.4 - 3.1	0.38 - 0.2	0.17 - 0.07	6.5 - 2.6
Ceiba pentandra	147.7 - 52.4	0.24 - 0.04	0.49 - 0.04	141 - 37.9	170.3 - 43	39.6 - 12.3	0.27 - 0.06	19.3 - 3.1	0.09 - 0.07	0.29 - 0.08	13.4 - 5.9
Colophospermum mopane	11.8 - 6.5	0.37 - 0.07	0.26 - 0.05	21.5 - 5.8	145.2 - 10.2	53.6 - 10.4	0.37 - 0.07	29.5 - 4.4	0.39 - 0.13	0.18 - 0.05	13 - 7.5
Combretum hereroense	9.6 - 6.4	0.53 - 0.06	0.23 - 0.04	14.6 - 4	305.8 - 51.4	164.1 - 38	0.24 - 0.05	25.9 - 5.2	0.35 - 0.14	0.21 - 0.08	4.1 - 3.2
Detarium microcarpum	19.6 - 8.8	0.31 - 0.07	0.14 - 0.07	62.6 - 19.2	146.1 - 17.8	45.8 - 14.4	0.55 - 0.13	16.1 - 1.8	0.13 - 0.1	0.35 - 0.1	24.7 - 7
Khaya anthotheca	42.9 - 20.4	0.49 - 0.11	0.3 - 0.08	245.4 - 133.1	193.5 - 35.9	95 - 32.6	0.21 - 0.1	17.8 - 2.4	0.25 - 0.16	0.2 - 0.07	6.2 - 1.4
Khaya grandifoliola	35.1 - 23	0.54 - 0.08	0.27 - 0.07	262.9 - 136.9	206.6 - 33	113.6 - 27	0.19 - 0.05	17.3 - 2.2	0.18 - 0.18	0.28 - 0.12	3.8 - 1.6
Khaya senegalensis	35.9 - 30.4	0.43 - 0.13	0.3 - 0.08	101.7 - 61.2	175.8 - 52.4	78.5 - 33.9	0.27 - 0.12	20.1 - 4.7	0.23 - 0.12	0.27 - 0.1	7 - 3
Millettia thonningii	24 - 14.8	0.32 - 0.08	0.32 - 0.07	73.9 - 39.8	235.8 - 61.7	78.8 - 34.4	0.36 - 0.07	21.7 - 2.6	0.06 - 0.04	0.21 - 0.1	9.8 - 5.7
Nauclea diderrichii	56.2 - 24.3	0.41 - 0.09	0.3 - 0.07	210.8 - 91.8	178.1 - 42.9	70.9 - 17.7	0.29 - 0.1	22.2 - 7	0.45 - 0.12	0.12 - 0.04	3.9 - 1.5
Peltophorum africanum	33.1 - 18.3	0.49 - 0.05	0.24 - 0.06	83.3 - 26.1	159 - 62.8	77.8 - 30.9	0.27 - 0.06	18.1 - 1.7	0.12 - 0.09	0.22 - 0.11	3.3 - 0.9
Pithecelobium dulce	49.9 - 23.6	0.29 - 0.07	0.34 - 0.06	17.4 - 3.8	286.2 - 125.3	88.8 - 55.4	0.37 - 0.06	24.8 - 2.9	0.2 - 0.11	0.19 - 0.11	7.4 - 4.4
Prosopis africana	6 - 4.7	0.3 - 0.1	0.12 - 0.06	22.8 - 10.5	223.7 - 57	67.7 - 29.6	0.58 - 0.14	14.1 - 1.1	0.32 - 0.43	0.3 - 0.06	19.1 - 5.9
Pterocarpus erinaceus	2.8 - 4	0.29 - 0.11	0.09 - 0.06	14.2 - 19.3	216.8 - 80	61.7 - 26.9	0.62 - 0.14	15.3 - 1.9	0.25 - 0.3	0.36 - 0.26	14.1 - 3.4
Senna siamea	119.6 - 35.4	0.51 - 0.06	0.3 - 0.04	195.2 - 82	186 - 64	95.1 - 37.4	0.19 - 0.05	22.2 - 1.8	0.31 - 0.18	0.15 - 0.04	6.1 - 3.6
Terminalia glaucescens	4.7 - 4.1	0.35 - 0.12	0.16 - 0.08	13.1 - 10.3	206.9 - 67.6	74.5 - 33.4	0.49 - 0.13	15.7 - 3.8	0.21 - 0.16	0.33 - 0.21	18.6 - 4.4
Terminalia ivorensis	54 - 26.1	0.38 - 0.07	0.42 - 0.05	55.2 - 25.3	206 - 34.8	79.7 - 22.1	0.21 - 0.06	16.9 - 2.3	0.37 - 0.15	0.22 - 0.14	6 - 1.7
Terminalia schimperi	28.9 - 16.1	0.32 - 0.08	0.34 - 0.09	6.2 - 1.9	310.3 - 75.7	101.7 - 34.4	0.34 - 0.11	27.1 - 3.3	0.2 - 0.08	0.26 - 0.08	7.8 - 2.7
Terminalia superba	71.7 - 25.9	0.3 - 0.05	0.41 - 0.06	95 - 27.7	216.5 - 38.2	65.6 - 17.3	0.29 - 0.05	17.6 - 1.9	0.28 - 0.1	0.18 - 0.12	4 - 1.4
Tetrapleura tetraptera	18.9 - 19	0.35 - 0.13	0.31 - 0.1	83.4 - 61.6	257 - 115.7	80.7 - 33	0.34 - 0.12	20 - 5.4	0.26 - 0.18	0.32 - 0.1	10.3 - 7.7
Vachellia erioloba	9.8 - 5.8	0.26 - 0.09	0.37 - 0.08	5.1 - 4.5	152.9 - 30.6	42.4 - 9.5	0.37 - 0.06	23 - 2.6	0.23 - 0.15	0.19 - 0.06	14.3 - 4.7
Vachellia tortilis	71.9 - 22.9	0.27 - 0.05	0.34 - 0.04	9.8 - 2.8	187.1 - 49.4	50.8 - 17.7	0.38 - 0.08	37.8 - 3.8	0.23 - 0.07	0.11 - 0.06	14.1 - 5.8

Table S3 General linear mixed model results for the differences between the vegetation clusters in (a) leaf traits and (savanna, forest), and (b) root traits (dry savannah, humid savannah, dry forest, moist forest). The F- and p-values are given for the differences between the clusters. For the random effects, we report the standard deviation (which is a measure for the variability for each random effect added to the model). We considered the following leaf traits: leaf mass fraction (LMF), stem mass fraction (SMF), leaf size (LS), specific leaf area (SLA), leaf area ration (LAR). And, we considered the following root traits: root mass fraction (RMF), mean rooting depth (MRD), specific root length fraction of the first section of the root (SRL fr1), specific root length fraction of the third section of the root (SRL fr3), starch concentration of the first section of the root ([starch]).

**(a)**			Random effects (s.d.)		Fixed effect Cluster	
	Trans-formation	*n*	Block number	Species	F-value	p-value
**LMF**	-	464	0.01	0.07	285.7	< 0.001
**SMF**	ln	464	0.006	0.39	136.6	< 0.001
**LS**	ln	439	< 0.001	1.24	117.4	< 0.001
**SLA**	√	436	0.28	1.27	1148.6	< 0.001
**LAR**	√	436	0.28	0.61	1160.4	< 0.001

**(b)**			Random effects (s.d.)		Fixed effect Cluster	
	Trans-formation	*n*	Block number	Species	F-value	p-value
**RMF**	ln	238	0.06	0.19	169.2	< 0.001
**MRD**	√	238	< 0.001	0.37	847.2	< 0.001
**SRL fr1**	ln	230	0.09	0.31	111.6	< 0.001
**SRL fr3**	-	214	< 0.001	0.02	199.4	< 0.001
**[starch]**	ln	219	< 0.001	0.31	222.0	< 0.001

Table S4a Above-ground trait values per cluster (marginal mean and standard error). LMF is leaf mass fraction, SMF is stem mass fraction, LS is leaf size, SLA is specific leaf area, LAR is leaf area ratio. S and F are the savannah (lower cluster in figure 3a) and forest cluster (upper cluster in figure 3a), respectively.

Clusters	LMF	SMF	LS	SLA	LAR
**S**	0.29 – 0.02	0.21 – 0.03	29.0 – 11.48	182.7 – 11.84	53.2 – 3.57
**F**	0.40 – 0.02	0.31 – 0.03	82.6 – 28.64	214.7 – 11.29	83.2 – 3.98

Table S4b Below-ground trait values per cluster (marginal mean and standard error). RMF is root mass fraction, MRD is mean rooting depth, SRL fr1 is specific root length fraction of the first section of the root, SRL fr3 is specific root length fraction of the third section of the root, and [starch] is the starch concentration of the first section of the root. DS is the dry savannah cluster (the third cluster in figure 3b), HS is the humid savanna cluster (the fourth cluster in figure 3b), DF is the dry forest cluster (upper cluster in figure 3b), and MF is the moist forest cluster (the second cluster in figure 3b).

Clusters	RMF	MRD	SRL fr1	SRL fr3	[starch]
**DS**	0.40 – 0.04	28.8 – 2.06	0.24 – 0.05	0.16 – 0.02	12.2 – 2.15
**HS**	0.52 – 0.05	15.9 – 1.37	0.12 – 0.02	0.35 – 0.02	16.6 – 2.59
**DF**	0.27 – 0.02	20.2 – 1.09	0.14 – 0.02	0.25 – 0.01	6.0 – 0.67
**MF**	0.23 – 0.02	20.6 – 1.56	0.33 – 0.06	0.17 – 0.02	4.7 – 0.78

Notes S1 Alternative clustering options and cluster relationships to environmental variables

Clusters can be obtained visually from a dendrogram. However, the number of clusters (i.e. how many branches are cut) depends on the significant differences between trait averages in the clusters, their biological meaning, and the possibility to link these with environmental variables. To explore this question, we consider alternative clustering options to the one used in the main text (Fig. 2a and Fig. S1) and analyse differences in mean trait values among clusters (Fig. S2) and mean environmental variables among clusters (Fig. S3).

For the clusters based on above-ground traits (Fig. S1a), the first alternative clustering option (i.e. cutting one more branch) results in clusters that are not significantly different from each other (Fig. S2b). Therefore, we can conclude that the most meaningful classification is the one presented in the main text with two clusters (Fig 2a). This is further illustrated when comparing environmental differences, which disappear in the first alternative clustering option: the open canopy cluster [2] is more limited in water, has less vegetation cover, and endures higher levels of fire than the closed or closing canopy cluster [1] as hypothesized (Fig S3a). However, the open canopy cluster [2] has more soil resources (as indicated by CEC) than the closed or closing canopy clusters [1] (Fig S3a). An explanation for this contradiction can be found in the main text.

For the clusters based on below-ground traits (Fig. 2b and Fig. S1b), each of the three explored alternative clustering options results in significantly different clusters (Fig. S2d-f). The first alternative (Fig. S2d) necessitates removing *Vachellia tortilis* from the analysis (Fig. S1b), which is less desirable. The second alternative clustering option (Fig S2e) splits the original dry forest cluster (Fig. S2c [1]) into two clusters (Fig. S2e [1,2]), where the only difference occurs in SRL fraction 1 (Fig. S2e). These two dry forest clusters have significantly different NDVI (Fig. S3e), with the more open dry forest (i.e. lower NDVI) [2] having a lower SRL fraction 1. This is in line with our hypothesis that less light limited environments have less fine-roots near the soil surface. However, given that these two clusters are only different in the basis of one out of five traits, we consider this clustering less robust than the original one (Fig 2b). The third alternative clustering option splits the humid savanna cluster (Fig. S2c [4]) into two clusters (Fig. S2f [5,6]), but neither traits nor environmental variables are significantly different between these two clusters (Fig. S2f [5,6], S3f [5,6]). Therefore, we conclude that the clustering option presented in the main text with four clusters and including *Vachellia tortilis* is the most robust for this data set (Fig. 2b). In this clustering option, as hypothesized, the humid savanna cluster [4] receives more rain than the dry savanna cluster [3], and the moist forest cluster [2] receives more rain than the dry forest cluster [1] (Fig S3c). Vegetation cover and, by implication, light differs between the two savanna clusters, with the dry savanna cluster [3] being more open. The dry forest cluster [1] has more frequent fires than the dry savanna cluster [3], and the moist forest cluster [2] has the lowest CEC, while the humid savanna cluster [4] has the highest CEC (Fig. S3c).

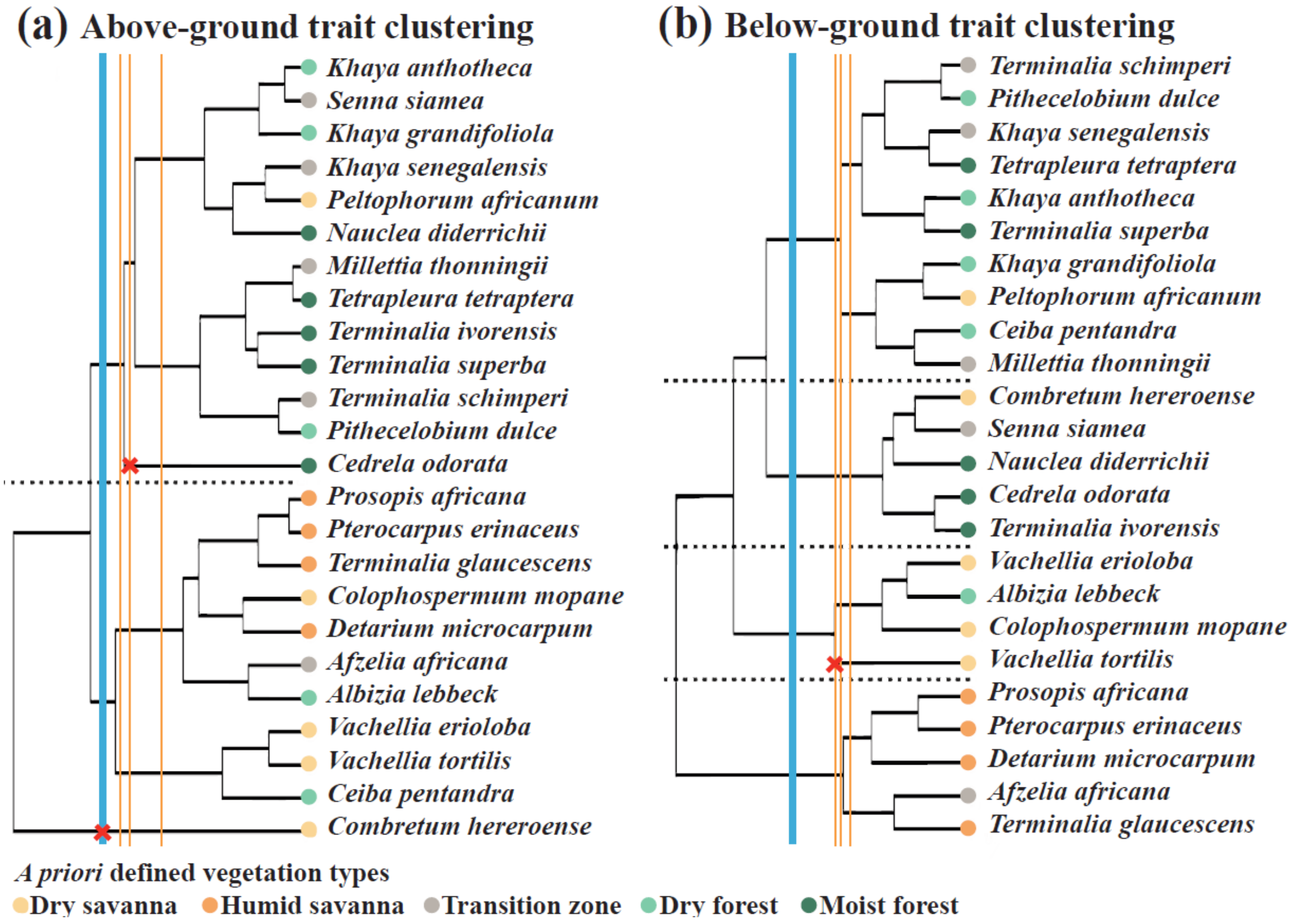

Figure S1 Overview of different clustering options for (a) above-ground trait clusters and (b) below-ground trait clusters. The blue line indicates the original clusters identified in the main text. The orange lines indicate three new clustering options explored further in figure S2 and S3.

Figure S2 (below) Trait averages of species clusters as identified in Figure S1. Bars represent marginal means and error bars show standard error. Small letters indicate significant differences ($p < 0.05$). The order of the bars (from left to right) follow that of the cluster diagram (from top to bottom). Colors indicate forest (green) and savanna (yellow) clusters as identified in the main text. The darker shade indicates the more humid vegetation type and the lighter shade indicates the dry vegetation type. Bars with the same color indicate that they were one cluster in a previous clustering option. (a,b) represent clusters based on above-ground traits, where (a) is the original clustering and (b) the first other clustering option (most left orange bar in Fig. S1a). *Combretum hereroense* was excluded from the analysis of these two clustering options, as it did not fit any of the two/three species clusters. (c-f) represent clusters based on below-ground traits, where (c) is the original clustering, (d) is the first other clustering option (most left orange bar in Fig. S1b), where merely *Vachellia tortilis* is removed from this and further clustering options as a one cluster species cannot be interpreted, (e) is the second other clustering option, and (f) is the third other clustering option indicated in Fig. S1b. Trait abbreviations: LMF = leaf mass fraction, SMF = stem mass fraction, LS = leaf size, SLA = specific leaf area, LAR = leaf area ratio, RMF = root mass fraction, MRD = mean rooting depth, SRL fraction 1 = specific root length fraction for the most upper section of the root, SRL fraction 3 = specific root length fraction for a deeper section of the root, [Starch] = starch concentration. The dashed line in the SRL fraction plots represents the null hypothesis of equal distribution of fine-roots over the whole length of the root, where asterisks indicate significant differences with this null hypothesis (** = $p < 0.01$, *** = $p < 0.001$).

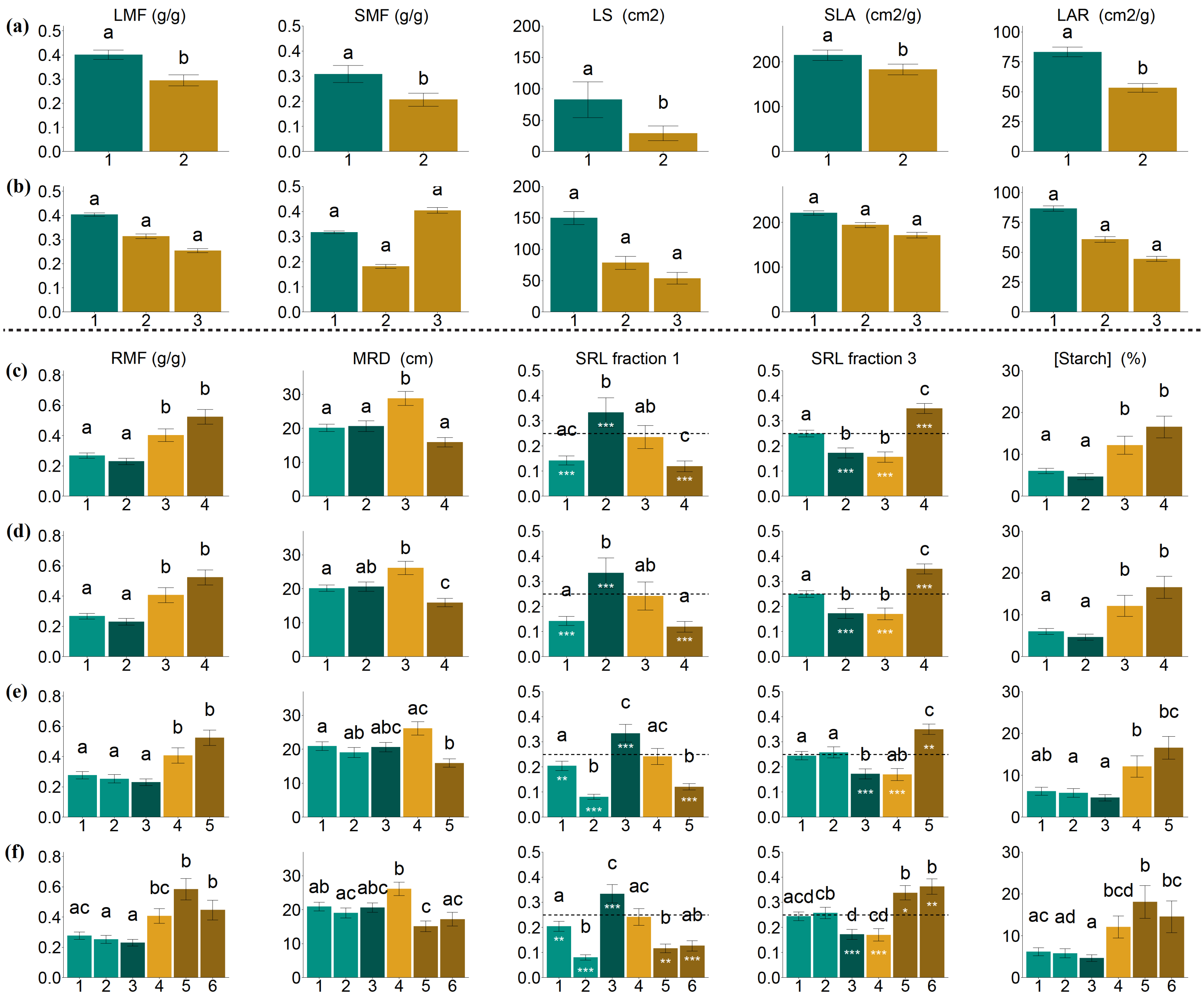

(a)
(b)
(c)
(d)
(e)
(f)
LMF (g/g)
SMF (g/g)
LS (cm2)
SLA (cm2/g)
LAR (cm2/g)
RMF (g/g)
MRD (cm)
SRL fraction 1
SRL fraction 3
[Starch] (%)

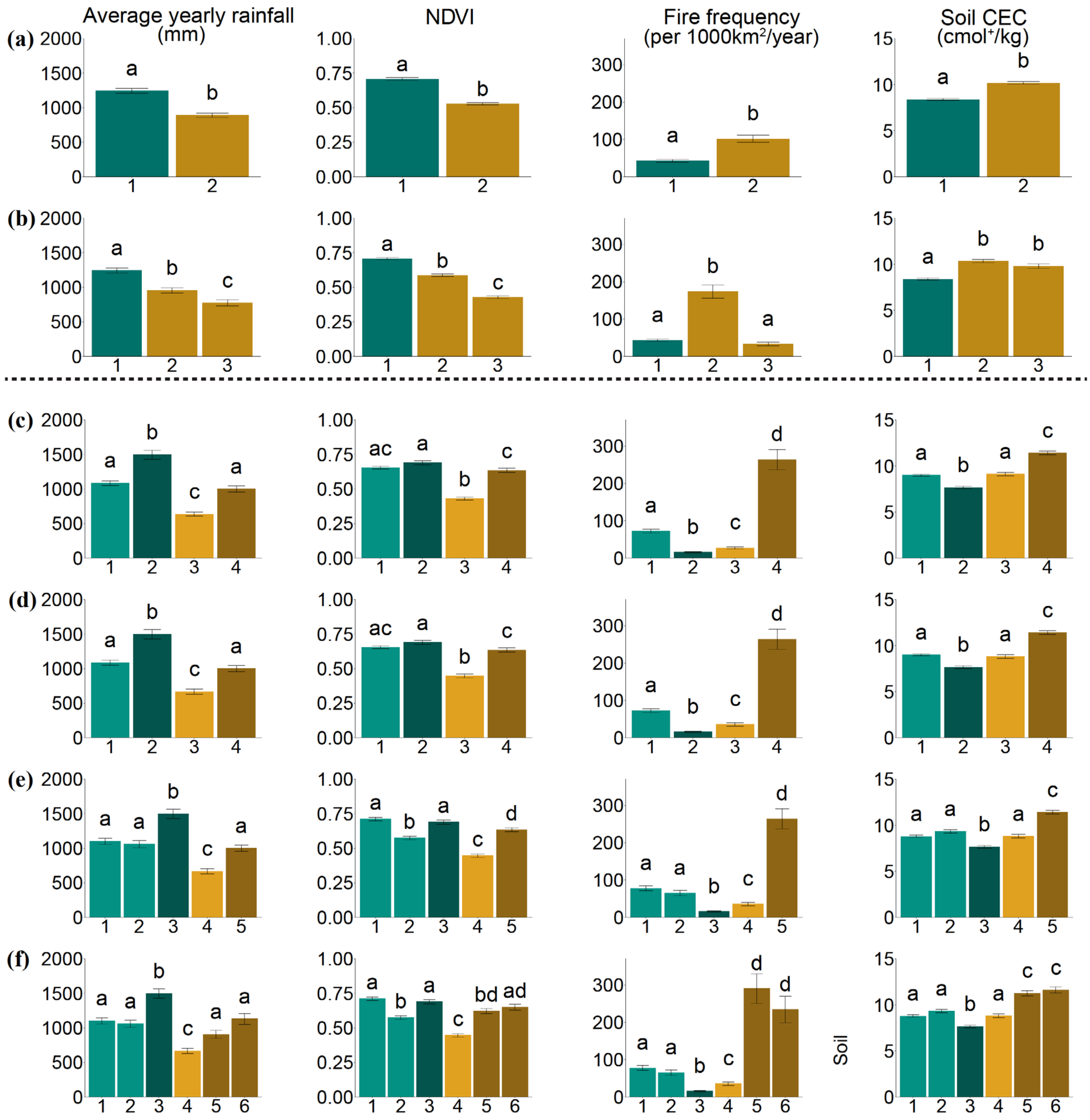

Figure S3 Environmental variable averages of species clusters as identified in Figure S1. Bars represent marginal means and error bars show standard error. Small letters indicate significant differences ($p < 0.05$). The order of the bars (from left to right) follow that of the cluster diagram (from top to bottom). Colors indicate forest (green) and savanna (yellow) clusters as identified in the main text. The darker shade indicates the more humid vegetation type and the lighter shade indicates the dry vegetation type. Bars with the same color indicate that they were one cluster in a previous clustering option. (a,b) represent environmental variable averages of clusters based on above-ground traits, where (a) is the original clustering and (b) the first other clustering option (most left orange bar in Fig. S1a). Environmental variable data of *Combretum hereroense* was excluded from the analysis of these two clustering options, as it did not fit any of the two/three species clusters. (c-f) represent environmental variable averages of clusters based on below-ground traits, where (c) is the original clustering, (d) is the first other clustering option (most left orange bar in Fig. S1b), where environmental variable data of *Vachellia tortilis* is removed from this and further clustering options as a one cluster species cannot be interpreted, (e) is the second other clustering option, and (f) is the third other clustering option indicated in Fig. S1b.
